# Supplementary material for: A fiber-deprived diet disturbs the fine-scale spatial architecture of the murine colon microbiome
Source: Nat Commun. 2019 Sep 25;10:4366. doi: 10.1038/s41467-019-12413-0 (PMC6761162; doi:10.1038/s41467-019-12413-0)
Supplement: Supplementary file 1 — Supplementary Information [file 41467_2019_12413_MOESM1_ESM.pdf]

Supplementary information for:

A fiber-deprived diet disturbs the fine-scale spatial architecture of the murine colon microbiome

Riva et al.

Supplementary information for:

A fiber-deprived diet disturbs the fine-scale spatial architecture of the murine colon microbiome

Alessandra Riva[1], Orest Kuzyk[1,‡], Erica Forsberg[2,3], Gary Siuzdak[2], Carina Pfann[1], Craig Herbold[1], Holger Daims[1], Alexander Loy[1], Benedikt Warth[2,4] and David Berry[1,5]

[1]Centre for Microbiology and Environmental Systems Science, Department of Microbiology and Ecosystem Science, Division of Microbial Ecology, University of Vienna, Althanstrasse 14, 1090 Vienna, Austria.

[2]The Scripps Research Institute, Scripps Center for Metabolomics and Mass Spectrometry, 10550 North Torrey Pines Road, La Jolla, California 92037, USA.

[3]Department of Chemistry and Biochemistry, San Diego State University, 5500 Campanile Drive, San Diego, California, 92182, USA.

[4]Department of Food Chemistry and Toxicology, University of Vienna, Währingerstraße 38, Vienna, Austria.

[5]Joint Microbiome Facility of the Medical University of Vienna and the University of Vienna, Vienna, Austria.

[‡]Deceased.

***Corresponding author:**

David Berry

Althanstrasse 14, 1090 University of Vienna, Austria

Phone: +43 1 4277 76612

berry@microbial-ecology.net

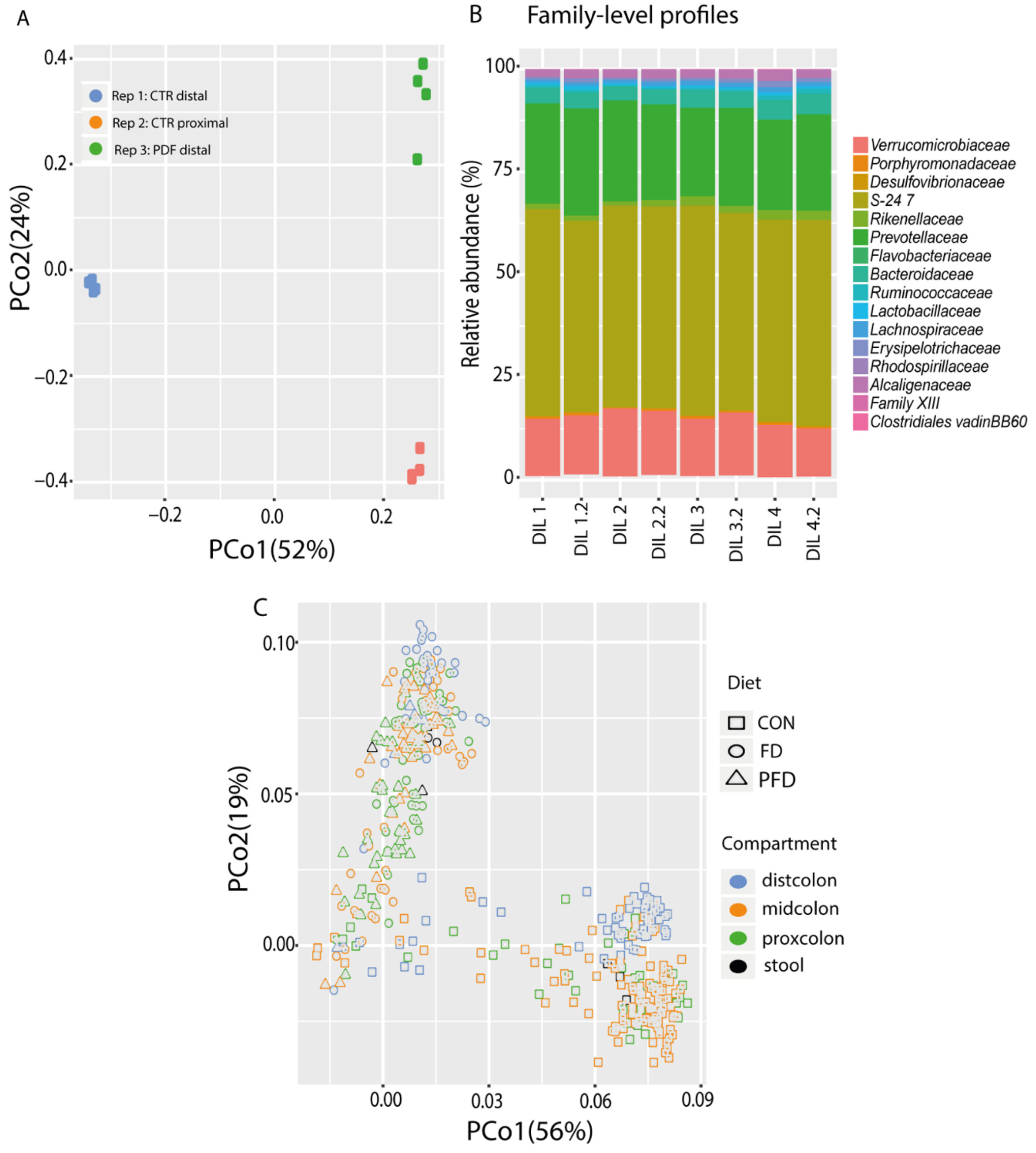

Supplementary Figure 1. **(A)** Principal component analysis (PCoA) shows the reproducibility of the LCM replicates perform in quadruplicate of different samples for different diet and location (CON distal, CON proximal, PFD distal). **(B)** Family-level profiles of 1:10 serial dilution performed in duplicates from a CON stool sample. The bar-plot shows the reproducibility of the duplicates even when diluted 10,000 times. (from 10 ng/ul to 0.001 ng/ul). We estimate that a typical laser capture microdissection sample would contain 1.7 ng/DNA, based on the sample volume (100 x100x10 µm=

10^5 μm^3) and assuming the sample volume consisted of cells of 1 μm^3 volume, each containing 0.017 pg of DNA. **(C)** PCoA of 16S rRNA gene amplicon sequence data displays grouping of samples by diet for all locations (stool, proximal, middle and distal colon) as well as stool.

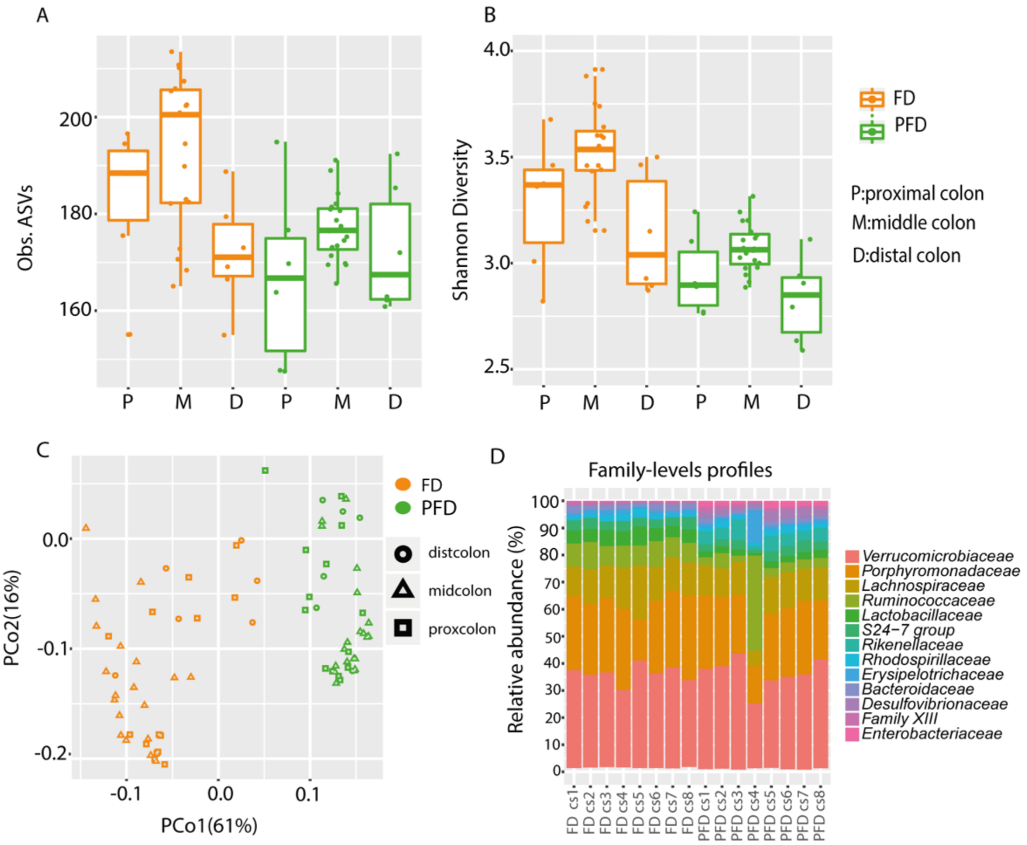

Supplementary Figure 2. 16S rRNA gene sequencing analysis of a separate experiment with independent mice (3 FD and 3 PFD diet). Alpha diversity **(A)** Observed species **(B)** Shannon diversity in FD and PFD diet (n=83, ANOVA FD vs. PFD $p<0.0001$ for both observed species and Shannon diversity. Boxplot: boxplot medians (center lines), interquartile ranges (box ranges), whisker ranges. Source data are provided as Source Data file. **(C)** Beta diversity shows significant grouping of the samples, confirmed by perMANOVA analysis (perMANOVA: diet p=0.001, longitudinal axis p=0.002 and lateral axis p=0.020). **(D)** Family-level composition of cross-sections along the proximal colon (from 1 to 8) in FD and PFD diet.

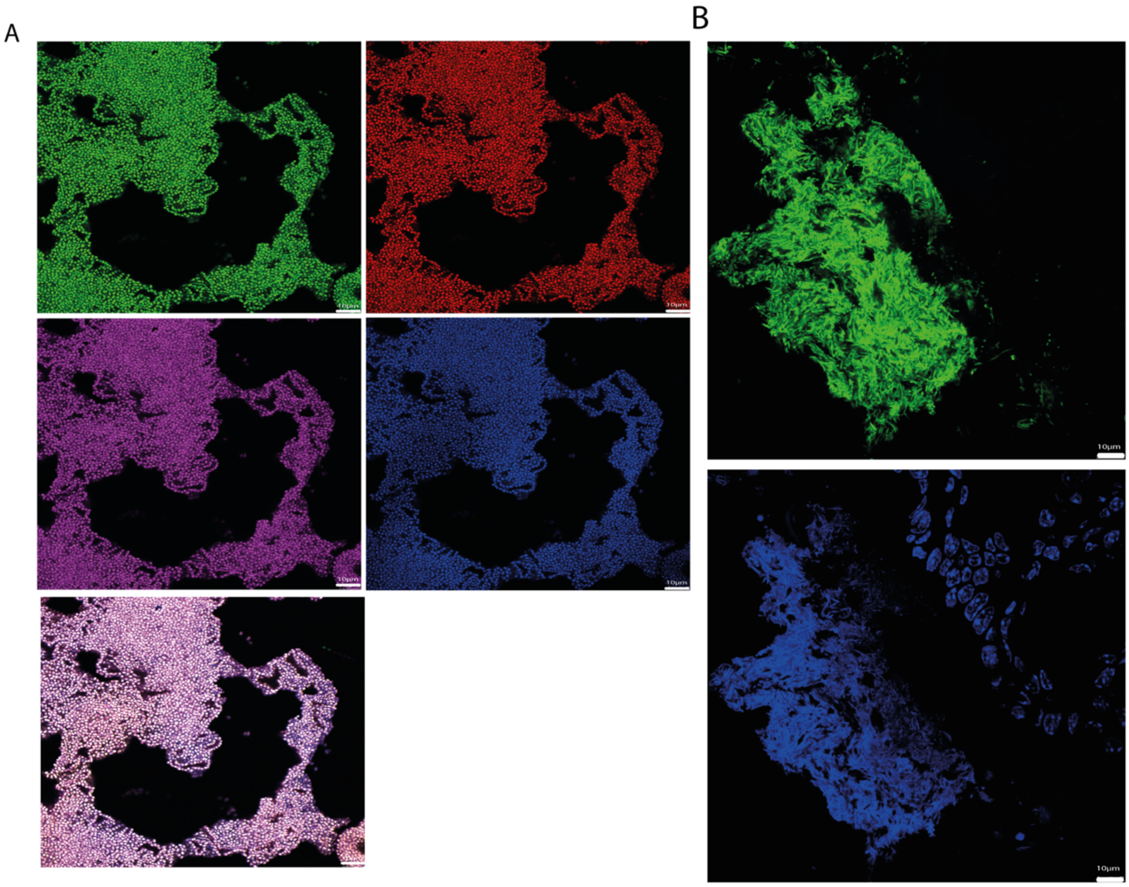

Supplementary Figure 3. **(A)** *Acinetobacter baumanni* stained with probes for all Bacteria (EUB 338 I-III; green), the genus-specific probe Aca 652 (red), *Gammaproteobacteria*-specific probe Gam42a (with unlabeled Bet42a competitor) (pink), and DAPI (blue). The overlay of all signals is shown on the bottom left. **(B)** Representative picture of a distal colon stained with the same set of probes show no Acinetobacter spp. signal.

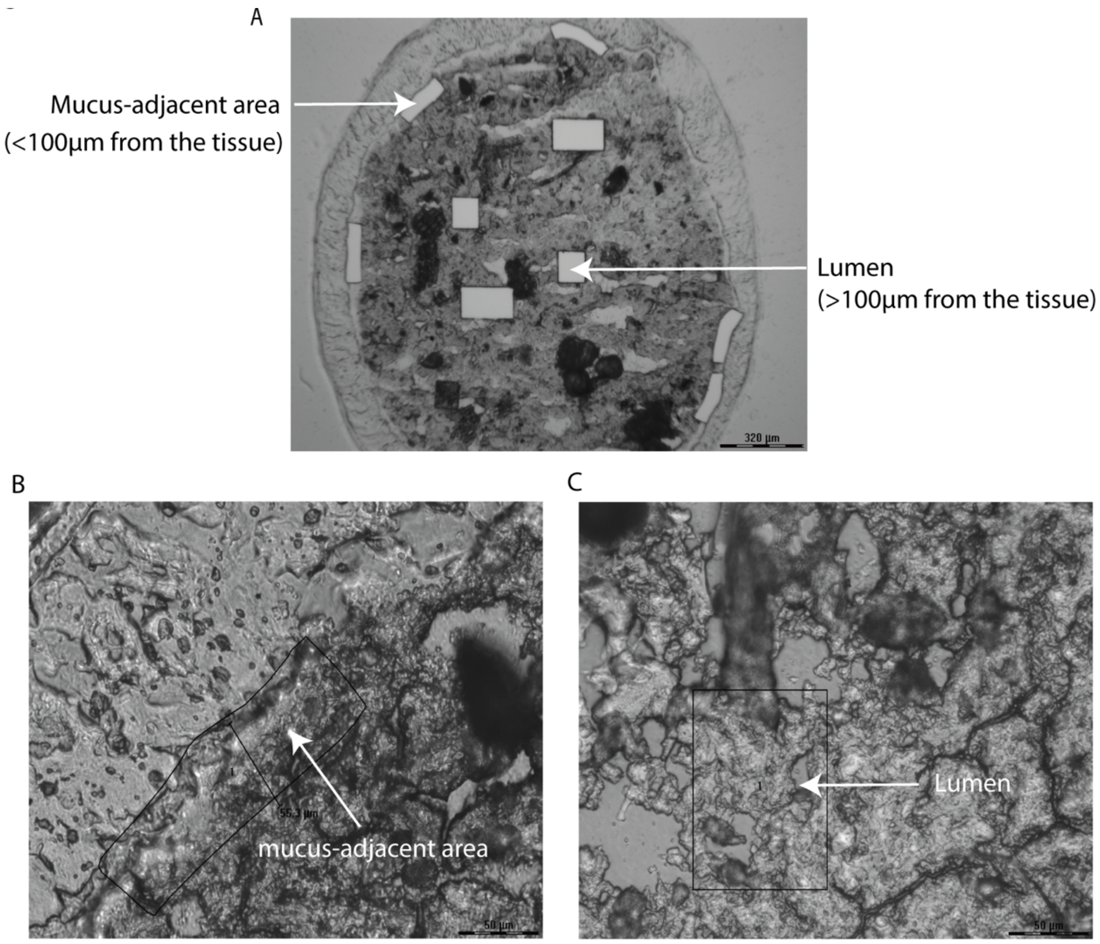

Supplementary Figure 4. (A) Sampling representation of a cryosection**.** Laser capture microdissection microscope was used to extract areas of approximately 100 x100 μm from mucosal and luminal content. At least 8 areas were sampled from each block (4 for mucus and 4 for lumen). **(B)** Mucus-adjacent areas (<100 μm from epithelial tissue) **(C)** lumen (>100 μm from epithelial tissue).

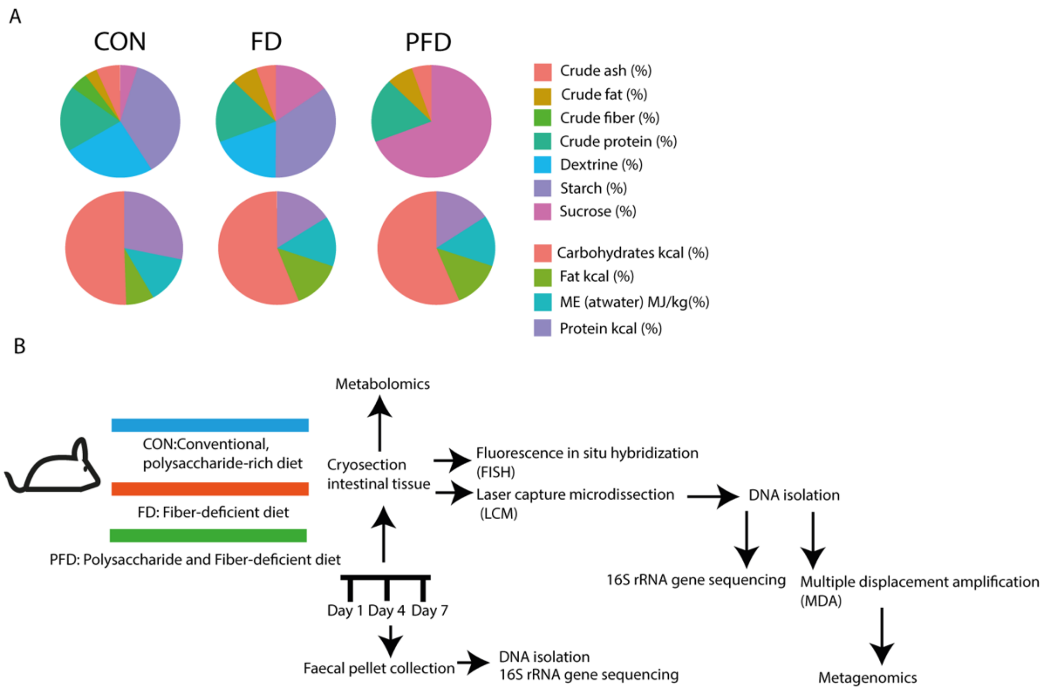

Supplementary Figure 5. Experimental design, sampling and analyses. **(A)**Three groups of mice were fed for 7 days with either control diet, fiber-deficient, or polysaccharide- and fiber-deficient diets. **(B)** Mice were sacrificed and the colon was divided into seven 1-cm blocks. Laser capture microdissection (LCM) was used to extract areas of approximately 100 x100 µm from both mucus-adjacent areas as well as areas distant from the tissue. At least 8 areas were sampled from each block (4 for mucus and 4 for lumen). DNA was extracted from laser micro-dissected samples and from fecal samples and the 16S rRNA gene was amplified for sequencing analysis. Additionally, metagenomic analysis of distal colon samples from CON and FD diets and global metabolomics on cecal samples were performed. Fluorescence in situ hybridization (FISH) and digital image analysis were carried out to target selected bacterial taxa at a single-cell level (Supplementary Table 1).

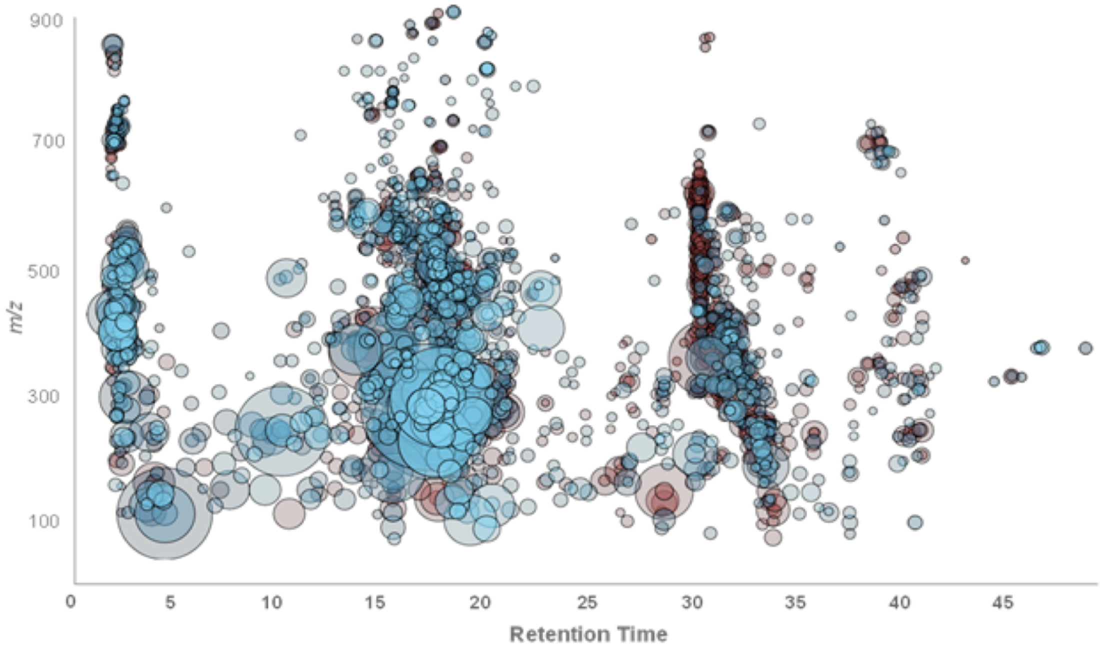

Supplementary Figure 6. Heterogeneity of metabolite patterns across mice fed with the different diets. The multi-group metabolomics cloud plot from XCMS Online illustrates 2,103 metabolic features significantly different (p-value ≤ 0.01, intensity > 10,000) between the three groups. Metabolic features are plotted m/z versus retention time along the HILIC gradient. The color of a bubble indicates the level of significance (p-value) with darker color representing more significant changes (thus lower p-values). The bubble size is an indicator of the ion intensity; the larger the bubble, the greater the relative change in metabolite concentration between diets. The most intense metabolic features were higher in the control diet, indicated by the blue color. Overall, more than half of the metabolic features were significantly altered between the diets highlighting the vast impact of fiber on gut microbial metabolism.

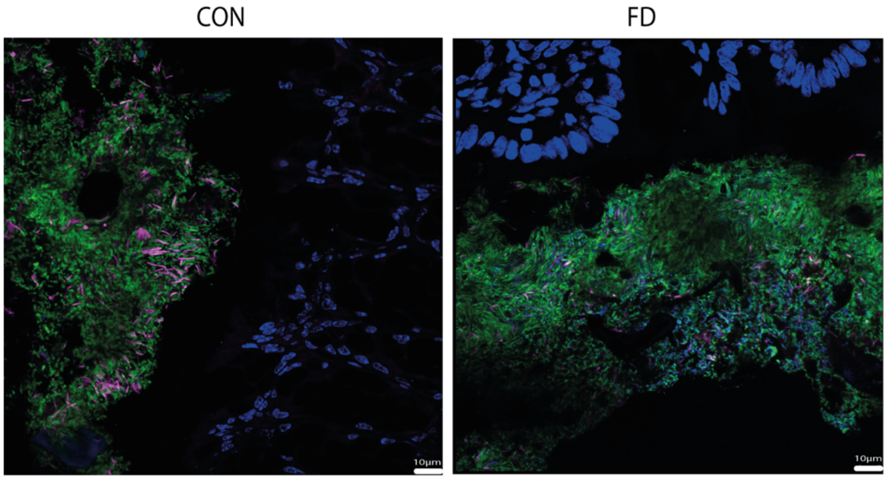

Supplementary Figure 7. Representative FISH image of a distal colon section in the conventional diet (CON) and in the fiber-deficient diet (FD). The pictures show the *Lachnospiraceae* stained with the specific probes Erec 482 (pink), all bacteria stained with EUB 338 I-III (green), and DNA stained with DAPI (blue).

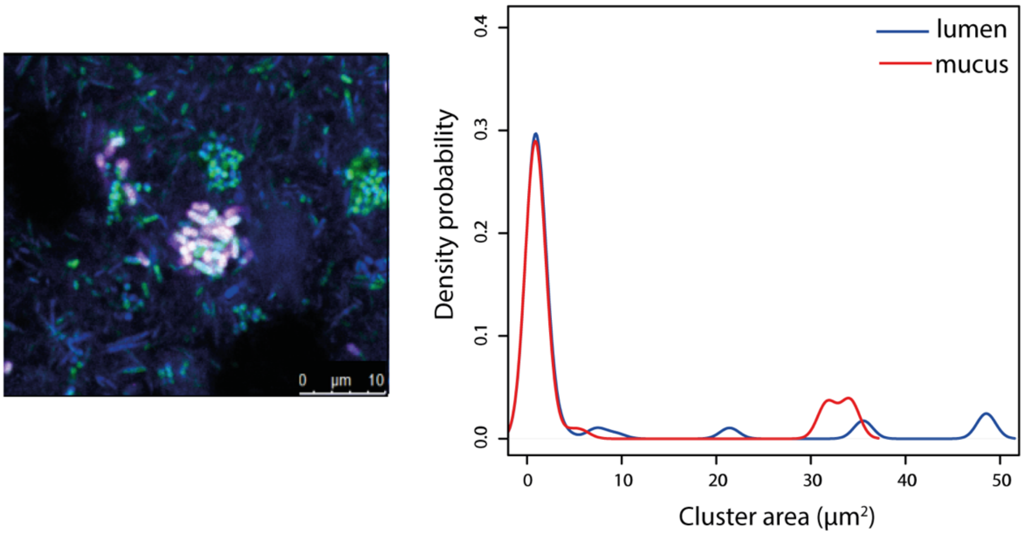

Supplementary Figure 8. FISH quantification of patchiness of *Desulfovibrio* species. The density probability with respect to cluster area (μm^2) in both lumen and mucus is shown (n=430). A representative FISH image shows a typical aggregation pattern in the distal colon in PFD diet. Sample was stained with a probe for *Desulfovibrio* spp. (Dsp_653; pink), a probe set for all Bacteria (EUB 338 I-III; green), and DAPI DNA stain (blue).

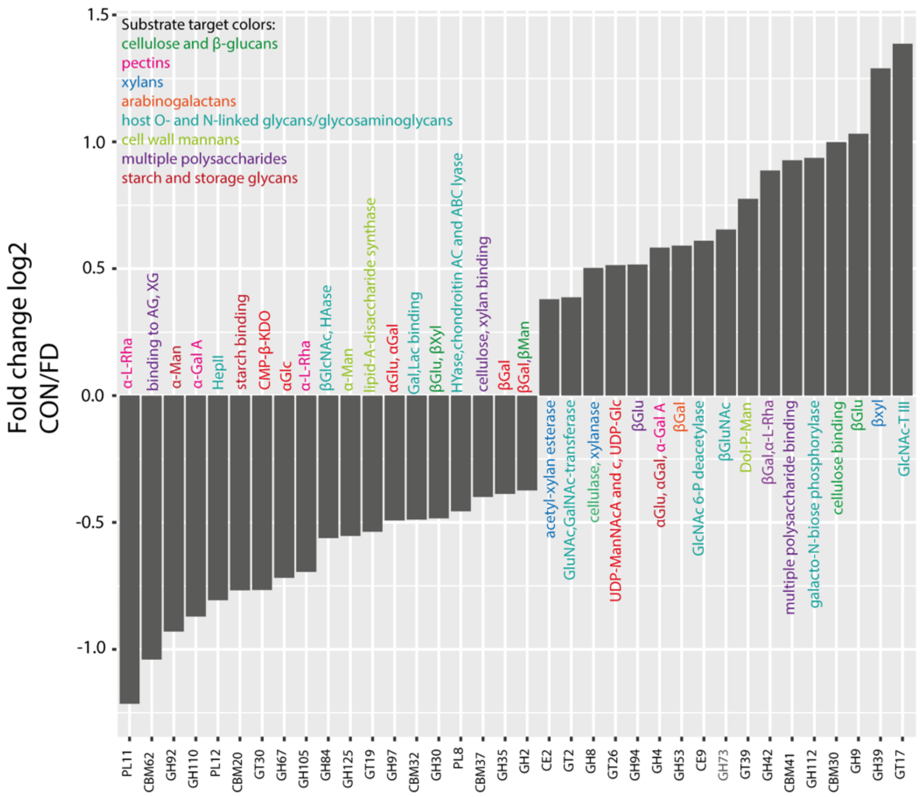

Supplementary Figure 9. Diet-specific changes in carbohydrate active enzyme genes. Positive and negative fold-changes in gene richness per CAZY category was calculated as log-2 normalized fold changes (CON/FD) of significant gene families ($p<0.05$).

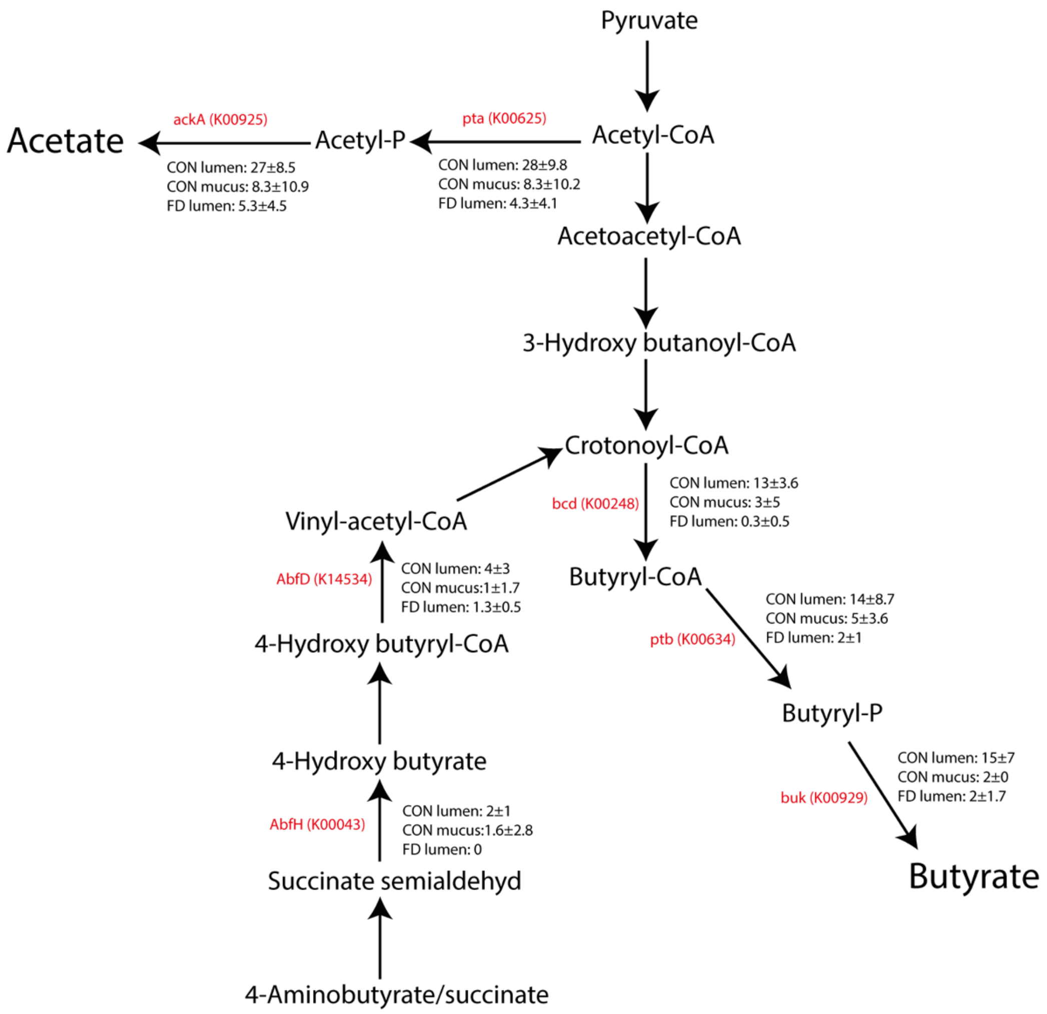

Supplementary Figure 10. Pathways for butyrate and acetate synthesis and corresponding genes (enzyme names) are displayed. FD changes in specific gene richness are indicated in red. *ackA* (acetate kinase), *pta* (phosphate acetyltransferase), *bcd* (butyryl-CoA dehydrogenase), *ptb* (phosphate butyryltransferase), *buk* (butyrate kinase), *abfD* (4-hydroxybutanoyl-CoA dehydratase), *abfH* (4-hydroxybutyrate dehydrogenase).

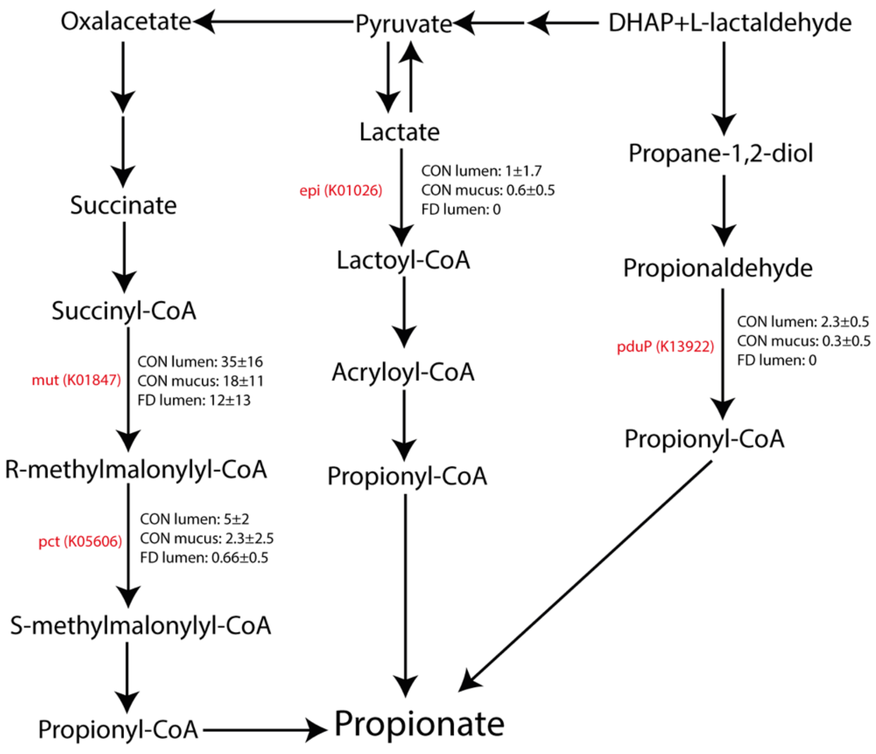

Supplementary Figure 11. Pathways for propionate synthesis (succinate pathway, acrylate pathway, propanediol pathways) and corresponding genes (enzyme names) are displayed. FD changes in specific gene richness are indicated in red. DHAP (dihydroxyacetonephosphate), mut (methylmalonyl coa mutase), *epi* (methylmalonyl coa epimerase), *pct* (propionate CoA-transferase), *pduP* (propionaldeihyde dehydrogenase).

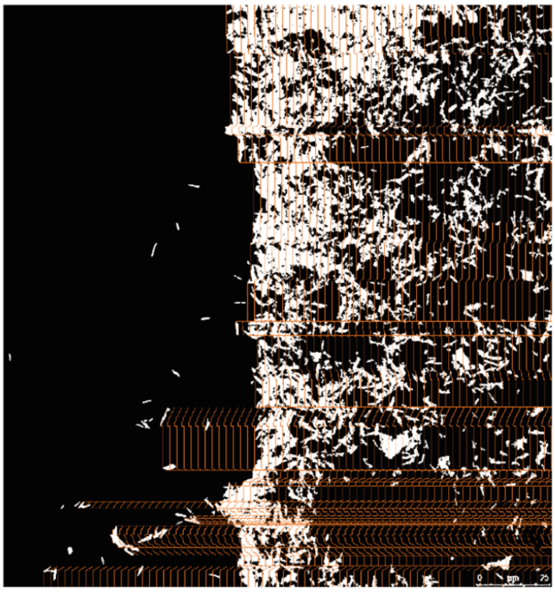

Supplementary Figure 12. Representation of a sliced image of the mucus area. Images were divided into 2 μm-thick sections from 0 to 150 μm starting from the mucus layer and then the biovolume fraction was calculated for each section as described in Methods.

Supplementary Table 1. List of rRNA-targeted oligonucleotide probes used for fluorescence in situ hybridization (FISH) analysis.

Probe name	specificity	probe sequence (5'->3')	fluorophore	FA (%)	References
Dsp653	*Desulfovibrio* species e.g. *piger*, *fairfieldensis*, *desulfuricans*, *intestinalis*, *oviles*, *legalii*, *simplex*	CCA CCC TCT CCC GGA TTC	Cy3	30	This study
Erec482	most of the *Clostridium coccoides-Eubacterium* rectale group (*Clostridium* cluster XIVa and XIVb)	GCT TCT TAG TCA RGT ACC G	Cy3 Dope[a]	0	[1]
ACA652	*Acinetobacter*	ATCCTCTCCCATAC TCTA	Cy3	35	[2]
Gam42a	*Gammaproteobacteria*	GCC TTC CCA CAT CGT TT	Cy5	35	[3]
Bet42a competitor	*Betaproteobacteria*	GCC TTC CCA CTT CGT TT	Cy3	35	[3]
EUB338-I	most Bacteria	GCTGCCTCCCGTAG GAGT	Fluos	0-50	[4]
EUB338-II	*Planctomycetales*	GCAGCCACCCGTA GGTGT	Fluos	0-50	[5]
EUB338-III	*Verrucomicrobiales*	GCTGCCACCCGTA GGTGT	Fluos	0-50	[5]
[b]Non-EUB	Complementary to EUB338 (Negative control)	ACTCCTACGGGAG GCAGC	-	0-50	[6]

[a]dope: double-fluorophore labeling of oligonucleotides.

[b]The NONEUB probe was used as a negative control for each experiment to test for non-specific binding of FISH probes.

Supplementary References

1. Franks A.H, Harmsen H.J, Raangs G.C, Jansen G.J, Schut F, and Welling G.W. Variations of bacterial populations in human feces measured by fluorescent in situ hybridization with group-specific 16S rRNA-targeted oligonucleotide probes. *Appl Environ Microbiol* **64**,3336–3345 (1998).
2. Wagner M, Erhart R, Manz W, Amann R, Lemmer H, Wedi D. and Schleifer K.H. Development of an rRNA-targeted oligonucleotide probe specific for the genus Acinetobacter and its application for in situ monitoring in activated sludge. *Appl Environ Microbiol* **60**,792-800 (1994).
3. Manz W, Amann R, Ludwig W, Wagner M, and Schleifer K.H. Phylogenetic oligodeoxynucleotide probes for the major subclasses of Proteobacteria-problems and solutions. *Syst Appl Microbiol* **15**,593–600 (1992).
4. Amann R.I, Binder B.J, Olson R.J, Chisholm S.W, Devereux R. and Stahl D. A. Combination of 16S rRNA-targeted oligonucleotide probes with flow cytometry for analyzing mixed microbial populations. *Appl Environ Microbiol* **56**,1919-1925 (1990).
5. Daims H, Brühl A, Amann R, Schleifer K.H, and Wagner M. The domain specific probe EUB338 is insufficient for the detection of all Bacteria: Development and evaluation of a more comprehensive probe set. *System Appl Microbiol* **22**,434-444 (1999).
6. Wallner G, Amann R. and Beisker W. Optimizing fluorescent in situ hybridization with rRNA-targeted oligonucleotide probes for flow cytometric identification of microorganisms. *Cytometry* **14**,136-143 (1993).
