# Supplementary material for: A fiber-deprived diet disturbs the fine-scale spatial architecture of the murine colon microbiome
Source: Nat Commun. 2019 Sep 25;10:4366. doi: 10.1038/s41467-019-12413-0 (PMC6761162; doi:10.1038/s41467-019-12413-0)
Supplement: Supplementary file 3 — Description of Additional Supplementary Files [file 41467_2019_12413_MOESM3_ESM.docx]

**Description of Supplementary Files**

**File Name: Supplementary Data 1**

**Description:** Metabolomic data shows more than 4,200 features detected by untargeted LC-MS analysis with over 2,100 being significantly different between the diet groups (p<=0.01, intensity threshold >10,000). Multiple comparison between diets are reported with p values.
