# Supplementary material for: A fiber-deprived diet disturbs the fine-scale spatial architecture of the murine colon microbiome
Source: Nat Commun. 2019 Sep 25;10:4366. doi: 10.1038/s41467-019-12413-0 (PMC6761162; doi:10.1038/s41467-019-12413-0)
Supplement: Supplementary file 5 — Reporting Summary [file 41467_2019_12413_MOESM5_ESM.pdf]

natureresearch

Corresponding author(s): David Berry

Reporting Summary

Nature Research wishes to improve the reproducibility of the work that we publish. This form provides structure for consistency and transparency in reporting. For further information on Nature Research policies, see Authors & Referees and the Editorial Policy Checklist.

Statistical parameters

When statistical analyses are reported, confirm that the following items are present in the relevant location (e.g. figure legend, table legend, main text, or Methods section).

n/a	Confirmed	
☐	☒	The exact sample size (n) for each experimental group/condition, given as a discrete number and unit of measurement
☐	☒	An indication of whether measurements were taken from distinct samples or whether the same sample was measured repeatedly
☐	☒	The statistical test(s) used AND whether they are one- or two-sided<br>*Only common tests should be described solely by name; describe more complex techniques in the Methods section.*
☐	☒	A description of all covariates tested
☐	☒	A description of any assumptions or corrections, such as tests of normality and adjustment for multiple comparisons
☐	☒	A full description of the statistics including central tendency (e.g. means) or other basic estimates (e.g. regression coefficient) AND variation (e.g. standard deviation) or associated estimates of uncertainty (e.g. confidence intervals)
☐	☒	For null hypothesis testing, the test statistic (e.g. F, t, r) with confidence intervals, effect sizes, degrees of freedom and P value noted<br>*Give P values as exact values whenever suitable.*
☒	☐	For Bayesian analysis, information on the choice of priors and Markov chain Monte Carlo settings
☒	☐	For hierarchical and complex designs, identification of the appropriate level for tests and full reporting of outcomes
☒	☐	Estimates of effect sizes (e.g. Cohen's d, Pearson's r), indicating how they were calculated
☐	☒	Clearly defined error bars<br>*State explicitly what error bars represent (e.g. SD, SE, CI)*

Our web collection on statistics for biologists may be useful.

Software and code

Policy information about availability of computer code

Data collection	No specialized data was used for data collection. All sequencing data has been deposited in public repositories.
Data analysis	All sequencing analyses were performed in the software R, as described in the Materials and Methods. Image analysis and metabolomics analysis weree also performed with frelly-available software as described in the Materials and Methods.

For manuscripts utilizing custom algorithms or software that are central to the research but not yet described in published literature, software must be made available to editors/reviewers upon request. We strongly encourage code deposition in a community repository (e.g. GitHub). See the Nature Research guidelines for submitting code & software for further information.

Data

Policy information about availability of data

All manuscripts must include a data availability statement. This statement should provide the following information, where applicable:

- Accession codes, unique identifiers, or web links for publicly available datasets
- A list of figures that have associated raw data
- A description of any restrictions on data availability

Sequencing data is available at the NCBI short read archive under SRP111824 (for amplicon data) and SRP128037 (for metagenomic data), metabolomics data under https://xcmsonline.scripps.edu/landing_page.php?pgcontent=listPublicShares, accession number 1153852 and 1175182.

Field-specific reporting

Please select the best fit for your research. If you are not sure, read the appropriate sections before making your selection.

☒ Life sciences ☐ Behavioural & social sciences

For a reference copy of the document with all sections, see nature.com/authors/policies/ReportingSummary-flat.pdf

Life sciences

Study design

All studies must disclose on these points even when the disclosure is negative.

Sample size	3 animals were used for each condition. At least 8 longitudinal sections were taken from each animal, and at least 4 mucus-adjacent and 4 lumen samples were taken from each section. The numbers were chosen based on feasibility, as suitable sample size calculations were not available.
Data exclusions	Data was excluded from amplicon sequencing datasets if operational taxonomic units were more abundant in negative controls, as described in the Materials and Methods.
Replication	Biological replicates (i.e. multiple animals) were included in the study to ensure reproducibility.
Randomization	Allocation was random.
Blinding	Blinding was not relevant to the study as we worked with mice and investigator knowledge is not expected to bias the outcome.

Materials & experimental systems

Policy information about availability of materials

n/a	Involved in the study	
☒	☐	Unique materials
☐	☐	Antibodies
☒	☐	Eukaryotic cell lines
☐	☒	Research animals
☒	☐	Human research participants

Antibodies

Antibodies used	*Describe all antibodies used in the study; as applicable, provide supplier name, catalog number, clone name, and lot number.*
Validation	*Describe the validation of each primary antibody for the species and application, noting any validation statements on the manufacturer's website, relevant citations, antibody profiles in online databases, or data provided in the manuscript.*

Research animals

Policy information about studies involving animals; ARRIVE guidelines recommended for reporting animal research

Animals/animal-derived materials	Nine wild-type C57BL/6 mice, 6-8 weeks of age, were used for the study.

Method-specific reporting

n/a	Involved in the study	
☒	☐	ChIP-seq
☒	☐	Flow cytometry
☒	☐	Magnetic resonance imaging
